# Editorial: Highlights in connected health 2021/22

**DOI:** 10.3389/fdgth.2022.1066860

**Published:** 2022-11-23

**Authors:** Pradeep Nair, Panagiotis Evaggelos Antoniou, Esteban J. Pino, Giuseppe Fico

**Affiliations:** ^1^ Department of New Media, Central University of Himachal Pradesh, Dharamshala, India; ^2^ Lab of Medical Physics, Medical School of Aristotle University of Thessaloniki, Thessaloniki, Greece; ^3^ Electrical Engineering Department, Universidad de Concepcion, Concepcion, Chile; ^4^ Department of Biomedical Engineering, Polytechnic University of Madrid, Madrid, Spain

**Keywords:** connected health, healthcare, digital health technologies, health solutions, patient access, patient empowerment, patient centricity

**Editorial on the Research Topic** Highlights in connected health 2021/22

The devastating impact of Covid-19 has compelled the healthcare leaders around the world to revisit the current healthcare systems in terms of response to the crisis in the new post-pandemic reality. It requires the redesign of healthcare systems by introducing new care models to address primary, secondary and acute care. Patients are demanding continuous access to healthcare services in a safe and convenient way and also demand a more agile, inter-professional delivery of care with empowered frontline staff leveraging technology. This further need a new framework to enable healthcare organizations to orchestrate the myriad interconnected changes required to sustain virtual care. The framework needs clinical and corporate strategies to ensure full connectivity between the core clinical workflows, supporting operations and technology platforms (1).

Here, connected health, a widely used term in public health studies, have the capability of providing cost-effective solutions at a time when the demands on healthcare services continue to increase due to the world's growing and ageing population, the rising costs of advanced medical treatments, and severely constrained healthcare budgets (2). Although the challenge is that the connected health solutions are technology-driven processes, hence, needs skill and willingness to engage with technology. This becomes crucial at a time, when the scale and pace of changes are high. Here the concerns are education and training. Education, training and widespread access to broadband technology can not only improve accessibility to connected health devices but can also be crucial in addressing gaps in our existing healthcare system (3). There is no doubt, that the technology has the power to improve access to healthcare services for people with mobility problems. Mobile technology is empowering patients and care givers by giving them more control over their health and making them less dependent on healthcare professionals for health information. With the help of digital technologies, they can not only search information online, but can also share their experiences and identify diagnostic and treatment options. An integrated healthcare system can exploit these technological opportunities further. Connecting patients to information, advice, and support can move the patient as a passive recipient of care to one where they are actively engaged in their own care (4).

Despite, that the connected health covers a large number of patient care points, the two central points witnessed across the globe are—patient access to care and patient empowerment. The connected health tools in recent times have not only improved patient care access and self-efficacy, but also delivered on central tenets of patient engagement. Connected health allows patients to connect with their medical providers more quickly and conveniently than ever before (5). The telehealth facilities allows patients to directly speak to their providers using video-conferences and at the same time it can connect the providers with one another to share consults, expertise, and knowledge during patient care. The benefits of connected health go beyond the logistic factors also—such as, it improves the way patients interact with and perceive their healthcare. When patients manage their own health using connected health tools, patients' empowerment and self-efficacy increase. It further put patients' in-charge of their own care. A fitness wearable helps a patient set her own fitness goals and track her progress towards those goals (6). A remote patient monitoring system alerts a diabetic patient when his sugars are too high, allowing the patient to make his own adjustments.

This research topic collection attempted to explore how the public healthcare has entered into a new phase of patient centricity, where the patients are becoming more and more responsible for their own healthcare thus creating enormous opportunities for engagement. The topic collection addressed the issues where the clinical and technology communities need to work closely to unlock the immense potential of connected health by leveraging technology supports to deliver cost-effective and quality care to everyone.

The published topic collection has studies having a diverse and vast coverage. The research article “*You are the on right track with the App:” Qualitative analysis of mobile phone use and user feedback regarding mobile phone sexual risk assessments for HIV prevention research” by Dietrich* et al*.* intends to understand participants mobile phone use to explore their perspectives on how to improve existing mobile application-based sexual risk assessment. The findings of the study provide user-centered approach to application design and development of conducting behavioural risk assessments in HIV prevention research. The study found that user recommendations are useful not only in improving mobile phone sexual risk assessment understandings but also for mHealth strategies used in HIV prevention programmes and research in South Africa. The study is relevant in optimizing the existing application-based mobile phone sexual risk assessments. A systematic review titled “*Digital predictors of morbidity, hospitalization, and mortality among older adults: a systematic review and metal analysis” by Daniolou* et al*.* provides evidence based understanding to synthesize and systematically analyze which digitally measureable data may be effectively collected through digital health devices to improve health outcomes for older people. While advocating leveraging of digital health tools to track parameters relevant to the health of old people, the study emphasizes on selecting an appropriate digitally measureable parameter to monitor the health conditions of old people and taking all the care to improve health outcomes among the elderly population. With the help of a modified PICO process and PRISMA (Preferred Reporting Items for Systematic Reviews and Meta-Analyses) framework, the study provides certain digitally measurable predictors of morbidity, hospitalization, and mortality among older adults aged 65 or older which can inform both technology developers and clinicians to plan for innovative design, development and clinical implementation of digital health technology applications for elderly citizens.

The review—“*Development of registry data to create interactive doctor-patient platforms for personalized patient care, taking the example of the DESTINY system” by Bergmann* et al*.* provides interesting insights into the effect of the integration of digital systems and their use in routine clinical care in outpatient medical practices—a niche area where there is less research available. The study suggests how to improve DESTINY (Database Assisted Therapy Decision Support System) to refine the existing components by integrating new modules thus making it more practical and user-friendly. Through the data collected and processed for the study, it provides certain ways to improve the care of individual patients and allows for a best possible usage of the healthcare systems' with limited human and non-human resources. Another review by *Hill* et al*. titled—“An integrative review on the feasibility and acceptability of delivering an online training and mentoring module to volunteers working in community organizations”* focused on the feasibility of online training programs for volunteers who support older adults. The review analyzed research literature in six databases and conducted an online search of online training programs currently being delivered in Canada. The review examined the feasibility and acceptability of community-based organizations adopting an online training format for their volunteers. The study encourages high engagement of volunteers by providing them timely mentorship and guidance. The study advocates that through sound technological and software interventions, online trainings can be designed more interactive and effective for the volunteers associated with community-based organizations.

A research study on “*Digital health coaching for type 2 diabetes: randomized controlled trail of healthy at home” by Azelton* et al*.* talks about the impact of *Health at Home*, a 12 week phone and SMS based digital health coaching program. The study compared the intervention to usual diabetic care in a family medicine residency clinic in a randomized controlled trail. The impact assessment of the study suggests that Healthy at Home digital health coaching slowed the natural progression of insulin resistance in T2DM. The findings of the study shows that the intervention has the ability to address SDOH and stage-match the intervention to the patients' level of motivation and literacy in order to overcome the barriers conventionally faced by the under-resourced. The study advocates that similar interventions implemented at the large scale can improve the long-term clinical efficacy, accessibility, scalability, and cost-effectiveness of comprehensive digital health coaching. The systematic review of the topic collection titled—*“the effectiveness of video animations as information tools for patients: a systematic review” by Byrne, T.M., Evans, E., Benhebil, N., & Knapp, P.* compares the effectiveness of video animations as information tools vs. other formats of delivery such as printed materials, verbal consultations or static images on patient knowledge, attitudes, cognitions, and behaviours. The study reviewed multiple databases and also undertook citation searching. The study used dual, independent decision-making for inclusion assessment, data extraction and quality appraisal. The findings were further reported through narrative synthesis. The findings of the study support video animation as a promising patient information tool for effects on knowledge.

We hope that this research topic collection will prove its worth for the readers by giving them some new insights on opportunities and challenges of connected healthcare. In this age of digital healthcare, we must acknowledge that the patients, caregivers and healthcare professionals should always be placed in the centre of connected health care in terms of both policy and practice approaches. It is very important to consider the needs, abilities, and constraints of them while designing and implementing connected healthcare services (7). While involving a lot of technologies and methods in the process, the role of social and cultural determinants of health should also be considered. There is no doubt that the future of healthcare relies on technology-enabled care options, but this also requires a transformative approach towards a world where patients and caregivers will feel more empowered in terms of access, care and comfort of cost-effective and quality healthcare services.

## Author contributions

The authors (s) confirm being the sole contributors of this work and have approved it for publication. All authors contributed to the article and approved the submitted version.

## References

[1] Kuziemsky C Abbas RM Carroll N. Toward a connected health delivery framework. *IEEE/ACM international workshop on software engineering in healthcare systems (SEHS)*. (2018). p. 46–9.

[2] Pattichis CS Panayides AS. Connected health. Front. Digit. Health. (2019) 1:1. 10.3389/fdgth.2019.00001 34713013 PMC8519500

[3] Karampela M Isomursu M Porat T Maramis C Mountford N Giunti G The extent and coverage of current knowledge of connected health: systematic mapping study. J Med Internet Res. (2019) 21(9):e14394. 10.2196/14394 31573915 PMC6785722

[4] Sannino G Pietro D Verde L. Healthcare systems: an overview of the most important aspects of current and future m-Health applications. In: El Saddik A Hossain M Kantarci B, editors. Connected health in smart cities. Cham: Springer (2020). 11 p. 10.1007/978-3-030-27844-1

[5] Simon P. The new paradigms of connected health—what impacts and effects on organizational models of care structures? In: Menvielle L Audrain-Pontevia AF Menvielle W, editors. The digitization of healthcare. London: Palgrave Macmillan (2017). 23 p. 10.1057/978-1-349-95173-4

[6] Jingshan L Pascale C. Health care 4.0: a vision for smart and connected health care. IISE Transa Healthc Syst Eng. (2021) 11(3):171–80. 10.1080/24725579.2021.1884627 PMC8423174 34497970

[7] Loncar TT Zdravevski E Machado DSJ Chouvarda I Trajkovik V. Literature on wearable technology for connected health: scoping review of research trends, advances, and barriers. J Med Internet Res. (2019) 21(9):e14017. 10.2196/14017 31489843 PMC6818529

